# 1,1′-{2,2′-[1,4-Phenyl­enebis(methyl­ene)]bis­(­oxy)bis­(2,1-phenyl­ene)}diethanone

**DOI:** 10.1107/S1600536811042528

**Published:** 2011-10-22

**Authors:** Nassir N. N. Al-Mohammed, Yatimah Alias, Zanariah Abdullah, Hamid Khaledi

**Affiliations:** a Department of Chemistry, University of Malaya, 50603 Kuala Lumpur, Malaysia

## Abstract

The asymmetric unit of the title compound, C_24_H_22_O_4_, contains one half-mol­ecule, the other half being generated by a crystallographic center of inversion. The central benzene ring makes a dihedral angle of 72.49 (5)° with the terminal benzene ring. In the crystal, adjacent mol­ecules are linked through C—H⋯O inter­actions, forming a sheet structure parallel to the *bc* plane. The sheets are stacked along the *a* axis *via* π–π inter­actions formed between the terminal benzene rings [centroid–centroid separation = 3.7276 (6) Å].

## Related literature

For related structures, see: Hu (2010 ▶); Tang *et al.* (2008 ▶).
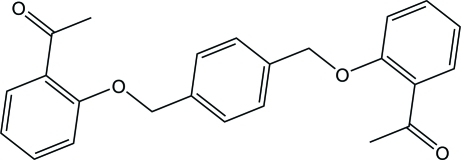


## Experimental

### 

#### Crystal data

C_24_H_22_O_4_
*M* *_r_* = 374.42
Orthorhombic, 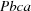

*a* = 6.8490 (1) Å
*b* = 15.0815 (2) Å
*c* = 17.8519 (3) Å
*V* = 1843.98 (5) Å^3^
*Z* = 4
Mo *K*α radiation
μ = 0.09 mm^−1^
*T* = 100 K
0.35 × 0.21 × 0.16 mm

#### Data collection

Bruker APEXII CCD diffractometer
Absorption correction: multi-scan (*SADABS*; Sheldrick, 1996 ▶) *T*_min_ = 0.969, *T*_max_ = 0.986
15454 measured reflections
2013 independent reflections
1728 reflections with *I* > 2σ(*I*)
*R*_int_ = 0.029

#### Refinement

*R*[*F* ^2^ > 2σ(*F* ^2^)] = 0.035
*wR*(*F* ^2^) = 0.100
*S* = 1.06
2013 reflections
128 parameters
H-atom parameters constrained
Δρ_max_ = 0.30 e Å^−3^
Δρ_min_ = −0.21 e Å^−3^

### 

Data collection: *APEX2* (Bruker, 2007 ▶); cell refinement: *SAINT* (Bruker, 2007 ▶); data reduction: *SAINT*; program(s) used to solve structure: *SHELXS97* (Sheldrick, 2008 ▶); program(s) used to refine structure: *SHELXL97* (Sheldrick, 2008 ▶); molecular graphics: *X-SEED* (Barbour, 2001 ▶); software used to prepare material for publication: *SHELXL97* and *publCIF* (Westrip, 2010 ▶).

## Supplementary Material

Crystal structure: contains datablock(s) I, global. DOI: 10.1107/S1600536811042528/is2792sup1.cif

Structure factors: contains datablock(s) I. DOI: 10.1107/S1600536811042528/is2792Isup2.hkl

Supplementary material file. DOI: 10.1107/S1600536811042528/is2792Isup3.cml

Additional supplementary materials:  crystallographic information; 3D view; checkCIF report

## Figures and Tables

**Table 1:** Hydrogen-bond geometry (Å, °)

*D*—H⋯*A*	*D*—H	H⋯*A*	*D*⋯*A*	*D*—H⋯*A*
C7—H7⋯O1^i^	0.95	2.56	3.4649 (14)	158

Symmetry code: (i) 
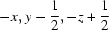
.

**Fig. 1.**
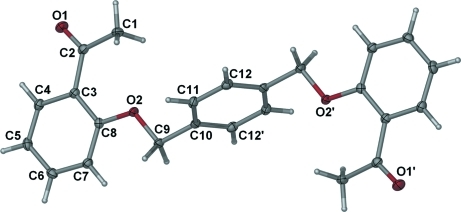
Molecular structure of the title compound with displacement ellipsoids drawn at 50% probability level. Hydrogen atoms are drawn as spheres of arbitrary radius. [Symmetry code: ' = -x, -y, -z.]

**Fig. 2.**
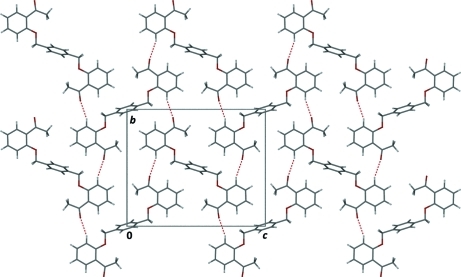
The two-dimensional network in the bc plane formed by C—H···O interactions (dashed lines).

## supplementary crystallographic information

### Comment

The title compound was obtained through the condensation of α,α'-dibromo-*p*-xylene with two equivalents of 2'-hydroxyacetophenone. The compound has a centrosymmetric molecular structure, the centroid of the central aromatic ring being located on an inversion center. The central aromatic ring makes a dihedral angle of 72.49 (5)° with the terminal rings. This value is comparable to those observed in similar structures (Hu, 2010; Tang *et al.*, 2008). In the crystal, the adjacent molecules are linked through C—H···O interactions (Table 1) to form a sheet parallel to the *bc* plane (Fig. 2). The sheets are connected into a three-dimensional network *via*π–π interactions formed between the terminal rings in the *a* direction [centroid-centroid separation = 3.7276 (6) Å].

### Experimental

To a suspension of α,α'-dibromo-*p*-xylene (1 g, 3.8 mmol) and potassium carbonate (1.05 g, 7.6 mmol) in dry acetone (25 ml), 2'-hydroxyacetophenone (1.03 g, 7.6 mmol) was added portionwise and the mixture was refluxed for 48 hr. The solvent was then evaporated under reduced pressure and the crude material was extracted by dichloromethane (3 × 25 ml). The combined organic layers was washed with water and brine and dried over anhydrous sodium sulfate. The solvent was evaporated under vacuum and the formed amorphous solid was re-crystallized from chloroform to obtain off-white crystals of the title compound (m.p. = 435–437 K).

### Refinement

Hydrogen atoms were placed at calculated positions and refined as riding atoms, with C—H distances of 0.95 (aryl), 0.98 (methyl) and 0.99 (methylene) Å, and with *U*_iso_(H) set to 1.2 (1.5 for methyl) *U*_eq_(C).

### Crystal data

**Table:**

C_24_H_22_O_4_	*F*(000) = 792
*M**_r_* = 374.42	*D*_x_ = 1.349 Mg m^−^^3^
Orthorhombic, *P**b**c**a*	Mo *K*α radiation, λ = 0.71073 Å
Hall symbol: -P 2ac 2ab	Cell parameters from 4755 reflections
*a* = 6.8490 (1) Å	θ = 2.7–30.5°
*b* = 15.0815 (2) Å	µ = 0.09 mm^−^^1^
*c* = 17.8519 (3) Å	*T* = 100 K
*V* = 1843.98 (5) Å^3^	Block, colorless
*Z* = 4	0.35 × 0.21 × 0.16 mm

### Data collection

**Table:**

Bruker APEXII CCD diffractometer	2013 independent reflections
Radiation source: fine-focus sealed tube	1728 reflections with *I* > 2σ(*I*)
graphite	*R*_int_ = 0.029
φ and ω scans	θ_max_ = 27.0°, θ_min_ = 2.7°
Absorption correction: multi-scan (*SADABS*; Sheldrick, 1996)	*h* = −8→8
*T*_min_ = 0.969, *T*_max_ = 0.986	*k* = −19→19
15454 measured reflections	*l* = −22→22

### Refinement

**Table:**

Refinement on *F*^2^	Primary atom site location: structure-invariant direct methods
Least-squares matrix: full	Secondary atom site location: difference Fourier map
*R*[*F*^2^ > 2σ(*F*^2^)] = 0.035	Hydrogen site location: inferred from neighbouring sites
*wR*(*F*^2^) = 0.100	H-atom parameters constrained
*S* = 1.06	*w* = 1/[σ^2^(*F*_o_^2^) + (0.0539*P*)^2^ + 0.5451*P*] where *P* = (*F*_o_^2^ + 2*F*_c_^2^)/3
2013 reflections	(Δ/σ)_max_ < 0.001
128 parameters	Δρ_max_ = 0.30 e Å^−^^3^
0 restraints	Δρ_min_ = −0.21 e Å^−^^3^

### Special details

**Table:**

Geometry. All e.s.d.'s (except the e.s.d. in the dihedral angle between two l.s. planes) are estimated using the full covariance matrix. The cell e.s.d.'s are taken into account individually in the estimation of e.s.d.'s in distances, angles and torsion angles; correlations between e.s.d.'s in cell parameters are only used when they are defined by crystal symmetry. An approximate (isotropic) treatment of cell e.s.d.'s is used for estimating e.s.d.'s involving l.s. planes.
Refinement. Refinement of *F*^2^ against ALL reflections. The weighted *R*-factor *wR* and goodness of fit *S* are based on *F*^2^, conventional *R*-factors *R* are based on *F*, with *F* set to zero for negative *F*^2^. The threshold expression of *F*^2^ > σ(*F*^2^) is used only for calculating *R*-factors(gt) *etc*. and is not relevant to the choice of reflections for refinement. *R*-factors based on *F*^2^ are statistically about twice as large as those based on *F*, and *R*- factors based on ALL data will be even larger.

### Fractional atomic coordinates and isotropic or equivalent isotropic displacement parameters (Å^2^)

**Table:**

	*x*	*y*	*z*	*U*_iso_*/*U*_eq_	
O1	0.04377 (13)	0.42646 (5)	0.16533 (5)	0.0229 (2)	
O2	0.04118 (12)	0.15272 (5)	0.15468 (4)	0.0159 (2)	
C1	0.07382 (18)	0.31010 (8)	0.07811 (6)	0.0203 (3)	
H1A	−0.0462	0.2780	0.0654	0.031*	
H1B	0.1854	0.2696	0.0751	0.031*	
H1C	0.0925	0.3591	0.0428	0.031*	
C2	0.05802 (16)	0.34623 (7)	0.15636 (6)	0.0165 (3)	
C3	0.05993 (16)	0.28692 (7)	0.22360 (6)	0.0146 (2)	
C4	0.06897 (16)	0.32882 (8)	0.29338 (6)	0.0171 (3)	
H4	0.0753	0.3917	0.2950	0.020*	
C5	0.06905 (17)	0.28253 (8)	0.35995 (7)	0.0187 (3)	
H5	0.0761	0.3130	0.4065	0.022*	
C6	0.05866 (16)	0.19058 (8)	0.35781 (6)	0.0177 (3)	
H6	0.0586	0.1579	0.4033	0.021*	
C7	0.04836 (16)	0.14611 (7)	0.29006 (6)	0.0160 (2)	
H7	0.0401	0.0832	0.2893	0.019*	
C8	0.05005 (15)	0.19348 (7)	0.22261 (6)	0.0140 (2)	
C9	0.02559 (17)	0.05749 (7)	0.15472 (6)	0.0162 (2)	
H9A	−0.0921	0.0387	0.1828	0.019*	
H9B	0.1417	0.0308	0.1789	0.019*	
C10	0.01168 (16)	0.02795 (7)	0.07449 (6)	0.0152 (2)	
C11	0.17455 (17)	0.03233 (7)	0.02807 (6)	0.0177 (3)	
H11	0.2946	0.0543	0.0472	0.021*	
C12	0.16313 (17)	0.00478 (7)	−0.04609 (6)	0.0172 (3)	
H12	0.2750	0.0083	−0.0774	0.021*	

### Atomic displacement parameters (Å^2^)

**Table:**

	*U*^11^	*U*^22^	*U*^33^	*U*^12^	*U*^13^	*U*^23^
O1	0.0305 (5)	0.0127 (4)	0.0254 (4)	0.0002 (3)	−0.0012 (4)	0.0010 (3)
O2	0.0239 (4)	0.0099 (4)	0.0138 (4)	−0.0011 (3)	−0.0001 (3)	−0.0015 (3)
C1	0.0278 (6)	0.0157 (5)	0.0176 (6)	−0.0019 (4)	−0.0012 (5)	0.0030 (4)
C2	0.0145 (5)	0.0141 (5)	0.0210 (6)	−0.0010 (4)	−0.0013 (4)	0.0016 (4)
C3	0.0131 (5)	0.0136 (5)	0.0171 (6)	0.0001 (4)	−0.0001 (4)	−0.0005 (4)
C4	0.0172 (5)	0.0133 (5)	0.0207 (6)	0.0000 (4)	−0.0002 (4)	−0.0032 (4)
C5	0.0186 (6)	0.0213 (6)	0.0162 (6)	−0.0008 (4)	−0.0001 (4)	−0.0056 (4)
C6	0.0174 (5)	0.0208 (6)	0.0150 (6)	−0.0007 (4)	0.0002 (4)	0.0019 (4)
C7	0.0165 (5)	0.0134 (5)	0.0182 (6)	−0.0001 (4)	−0.0005 (4)	0.0000 (4)
C8	0.0127 (5)	0.0147 (5)	0.0147 (5)	0.0003 (4)	−0.0004 (4)	−0.0022 (4)
C9	0.0229 (6)	0.0097 (5)	0.0159 (5)	0.0000 (4)	−0.0001 (4)	−0.0002 (4)
C10	0.0220 (6)	0.0082 (5)	0.0153 (5)	0.0018 (4)	−0.0010 (4)	−0.0005 (4)
C11	0.0177 (6)	0.0150 (5)	0.0204 (6)	−0.0019 (4)	−0.0020 (4)	−0.0019 (4)
C12	0.0191 (6)	0.0134 (5)	0.0193 (6)	−0.0001 (4)	0.0028 (4)	−0.0008 (4)

### Geometric parameters (Å, °)

**Table:**

O1—C2	1.2246 (13)	C6—C7	1.3846 (15)
O2—C8	1.3609 (13)	C6—H6	0.9500
O2—C9	1.4401 (12)	C7—C8	1.4002 (15)
C1—C2	1.5033 (15)	C7—H7	0.9500
C1—H1A	0.9800	C9—C10	1.5029 (15)
C1—H1B	0.9800	C9—H9A	0.9900
C1—H1C	0.9800	C9—H9B	0.9900
C2—C3	1.4970 (15)	C10—C12^i^	1.3907 (16)
C3—C4	1.3982 (15)	C10—C11	1.3912 (16)
C3—C8	1.4109 (15)	C11—C12	1.3897 (16)
C4—C5	1.3783 (16)	C11—H11	0.9500
C4—H4	0.9500	C12—C10^i^	1.3908 (16)
C5—C6	1.3892 (16)	C12—H12	0.9500
C5—H5	0.9500		
			
C8—O2—C9	116.96 (8)	C6—C7—C8	120.24 (10)
C2—C1—H1A	109.5	C6—C7—H7	119.9
C2—C1—H1B	109.5	C8—C7—H7	119.9
H1A—C1—H1B	109.5	O2—C8—C7	122.38 (9)
C2—C1—H1C	109.5	O2—C8—C3	117.68 (9)
H1A—C1—H1C	109.5	C7—C8—C3	119.95 (9)
H1B—C1—H1C	109.5	O2—C9—C10	107.45 (8)
O1—C2—C3	119.09 (10)	O2—C9—H9A	110.2
O1—C2—C1	119.05 (10)	C10—C9—H9A	110.2
C3—C2—C1	121.86 (9)	O2—C9—H9B	110.2
C4—C3—C8	117.69 (10)	C10—C9—H9B	110.2
C4—C3—C2	116.40 (10)	H9A—C9—H9B	108.5
C8—C3—C2	125.90 (10)	C12^i^—C10—C11	119.34 (10)
C5—C4—C3	122.64 (10)	C12^i^—C10—C9	120.48 (10)
C5—C4—H4	118.7	C11—C10—C9	120.18 (10)
C3—C4—H4	118.7	C12—C11—C10	120.54 (11)
C4—C5—C6	118.80 (10)	C12—C11—H11	119.7
C4—C5—H5	120.6	C10—C11—H11	119.7
C6—C5—H5	120.6	C11—C12—C10^i^	120.12 (11)
C7—C6—C5	120.68 (10)	C11—C12—H12	119.9
C7—C6—H6	119.7	C10^i^—C12—H12	119.9
C5—C6—H6	119.7		

Symmetry codes: (i) −*x*, −*y*, −*z*.

### Hydrogen-bond geometry (Å, °)

**Table:**

*D*—H···*A*	*D*—H	H···*A*	*D*···*A*	*D*—H···*A*
C7—H7···O1^ii^	0.95	2.56	3.4649 (14)	158.

Symmetry codes: (ii) −*x*, *y*−1/2, −*z*+1/2.
